# Editorial: Health and Performance Assessment in Winter Sports

**DOI:** 10.3389/fspor.2021.628574

**Published:** 2021-03-09

**Authors:** Jörg Spörri, Thomas Stöggl, Kamiar Aminian

**Affiliations:** ^1^Sports Medical Research Group, Department of Orthopaedics, Balgrist University Hospital, University of Zurich, Zurich, Switzerland; ^2^University Centre for Prevention and Sports Medicine, Department of Orthopaedics, Balgrist University Hospital, University of Zurich, Zurich, Switzerland; ^3^Department of Sport Science and Kinesiology, University of Salzburg, Hallein, Austria; ^4^Red Bull Athlete Performance Centre, Thalgau, Austria; ^5^Laboratory of Movement Analysis and Measurement, Ecole Polytechnique Fédérale de Lausanne, Lausanne, Switzerland

**Keywords:** algorithms, biomechanics, diagnostics, digital health, injury prevention, performance enhancement, sensors, athlete

Recent developments in technology and engineering have provided novel solutions for monitoring health and performance, as well as assessing key variables that would not have been easily accessible a few years ago. Options include digital solutions for collecting self-reported data on physical activity, recovery, psychological readiness, or injury (Düking et al., 2018), measurement technologies for quantifying physiological variables (Khundaqji et al., 2020), wearable sensors for motion analysis (Sperlich et al., 2020), and technology-based approaches for performance and load quantification (Lutz et al., 2019). Moreover, customized algorithms and data analytics help to extract and visualize relevant metrics for effective coaching and athletes' health protection (Rommers et al., 2020).

A close relationship between engineers, coaches, sports scientists, and medical professionals ensures the success of healthy sporting activity and the sustainable long-term development of athletes throughout their careers. This is especially true for winter sports and youth athletes. On the one hand, the assessment of health and performance in winter sports is a permanent challenge and, from a technological point of view, particularly difficult. Winter sports take place under extreme and hardly standardisable outdoor conditions. Therefore, various research is done in laboratory situations (e.g., ski simulators, ski ergometers, imitation movements of winter sports-specific motions, etc.). However, in many cases, there is an evident need to bring the lab to the field and assess health and performance-related aspects within representative real-life settings (Spörri et al., 2016). On the other hand, youth athletes need special care because they are particularly susceptible to the long-term consequences of sports participation and injuries during phases of biological maturation and rapid musculoskeletal growth (Schoeb et al., 2020). However, there is great potential in young athletes being more familiar with digitalization and consequently better able to use new technologies.

Accordingly, the main objective of our Research Topic entitled “Health and Performance Assessment in Winter Sports” was to emphasize the relationship between health, performance, and technology, and to highlight current challenges in the design of innovative measuring systems, wearable sensors, and assessment protocols for examining and monitoring health and performance in sports like Freestyle, Alpine, Nordic and Paralympic skiing. It was also intended to compile research articles focusing on the application of digitalization and technology in the context of performance enhancement, injury prevention, and rehabilitation.

## Current Trends of Health and Performance Assessment in Winter Sports

Recent advances in digitization and measurement technology have also influenced youth and elite winter sports. Current trends of health and performance assessment can be summarized as: (1) new technologies and approaches for motion analysis with a clear tendency toward wearables, large capture volumes and long-term assessments (Supej, 2010; Gilgien et al., 2013, 2015; Fasel et al., 2017a,b; Spörri et al., 2017; Fasel et al., 2018; Gløersen et al., 2018a,b; Takeda et al., 2019; Neuwirth et al., 2020); (2) digital data collections, interdisciplinary/integrative measurement setups, advanced signal processing and state-of-the-art data analytics such as machine learning in connection with performance assessment and enhancement (Rindal et al., 2017; Jang et al., 2018; Losnegard et al., 2019; Ostrek et al., 2019; Skattebo et al., 2019; Heinrich et al., 2020; Solli et al., 2020); (3) mobile Health (mHealth) applications, supporting engineering solutions, and technology-based athlete screening in the context of injury/illness surveillance and prevention (Steenstrup et al., 2018; Schindelwig et al., 2019; Ellenberger et al., 2020a,b; Franchi et al., 2020; Fröhlich et al., 2020; Hermann and Senner, 2020); and (4) comprehensive monitoring/test protocols, and the objectification of clinical criteria in the return-to-sport context (Jordan et al., 2015a,b; Jordan et al., 2018; Csapo et al., 2019).

Fully in line with these current trends, our Research Topic on “Health and Performance Assessment in Winter Sports” compromises a total of 13 research articles, all of which can be assigned to one of the trend areas mentioned above (see Table 1). Of these articles, 11 were original research articles, one a perspective and one a methods article. Most articles were cross-sectional observations, while there were only three articles based on other study designs (one case study, one intervention study, and one literature review).

**Table 1:** Overview of the published research articles categorized by area, winter sports, article type/study design, and subjects.

**Area**	**Winter sports**	**Article type/Study design**	**Subjects**	**References**
**New technologies and approaches**				
Gyroscopes for turn switch detection	Alpine skiing	Original research article/cross-sectional study	*n* = 11	Martinez, Brunauer et al.
Pressure insoles vs. force plates for turn switch detection	Alpine skiing	Original research/cross-sectional study	*n* = 20	Martinez, Nakazato et al.
GNSS for performance analysis (skier kinematics)	Alpine skiing	Perspective article/literature review	n/a	Supej et al.
**Performance assessment and enhancement**				
Discipline-dependent knee and hip joint kinematics using electro-goniometers	Alpine skiing	Original research article/cross-sectional study	*n* = 20	Alhammoud et al.
Level-, course and terrain-dependent performance analysis by GNSS	Alpine skiing	Original research article/cross-sectional study	*n* = 57	Bruhin et al.
Effect of sitting posture on sit-skiing economy based on an interdisciplinary/integrative measurement set-up	Paralympic Cross-Country skiing	Original research article/cross sectional study	*n* = 10	Lajunen et al.
Determinants of competition performance based on an interdisciplinary/integrative measurement set-up	Biathlon	Original research article/cross-sectional study	*n* = 11	Luchsinger et al.
Skier technique and tactic analysis by video-based 3D kinematics	Alpine skiing	Original research article/cross-sectional study	*n* = 6	Reid et al.
Relative age effect in youth competitive alpine skiers based on a comprehensive database	Alpine skiing	Original research article/cross-sectional study	*n* = 1,466	Steidl-Müller et al.
**Injury/illness surveillance and prevention**				
Self-reported physical activity, injury, and by using an online questionnaire	Alpine skiing	Original research article/cross-sectional study	*n* = 96	Doyle-Baker and Emery
Exercise in the cold with special focus on heat-and-moisture-exchanging breathing devices	Cross-Country skiing	Original research article/cross-sectional study	n/a	Hanstock et al.
Evaluation of a sports-specific injury prevention program based systematic injury monitoring system	Alpine skiing	Original research article/intervention study with historical controls	*n* = 736	Westin et al.
**Return-to-sport**				
Monitoring the return-to-sport transition after ACL injury by digital self-reporting and neuromuscular testing	Alpine skiing	Methods article/case study	*n* = 1	Jordan et al.

*GNSS, Global Navigation Satellite Systems; 3D, Three-dimensional; ACL, anterior cruciate ligament*.

With respect to new technologies and approaches, Martinez, Brunauer et al. developed and validated a gyroscope-based ski turn detection algorithm for an on-snow application under various conditions. For many health and performance-related applications in alpine skiing, an exact determination of separate turn cycles is key (Spörri et al., 2012, 2018). An alternative, vertical ground-reaction force(GRF)-based approach was investigated in Martinez, Nakazato et al. In their study, they compared different functional definition criteria, as well as portable force platforms vs. pressure insoles for the determination of turn switches during alpine skiing. Supej et al. summarized the current state of scientific knowledge on methodological and practical aspects of the assessment of alpine skiing performance by Global Navigation Satellite Systems (GNSS) and stimulated future perspectives on the topic. GNSS-based performance assessments have been widely used in research on alpine and cross-country skiers (Supej, 2010; Gilgien et al., 2013, 2015; Gløersen et al., 2018a), and many skiing teams already employ GNSS technology in their daily training and equipment testing routines. Thus, in combination with advanced computer software, they are a good example of how the digital revolution has also taken hold in winter sports.

With regard to performance assessment and enhancement, the current Research Topic offers a broad spectrum of digitalization and technology-driven sample applications in the fields of alpine skiing, cross-country skiing, and biathlon. Alhammoud et al. and Bruhin et al. for example, have used state-of-the-art wearable measurement technologies to analyse the racecourse (e.g., course setting, steepness, etc.) and motion patterns/performance characteristics (e.g., joint angles, speed, time per turn, turn phases, etc.) of competitive alpine skiers at all levels and within different alpine disciplines. Moreover, Reid et al. used a complex video-based 3D kinematic analysis to quantify the ski motion characteristics during alpine slalom skiing and compare these measures with theoretical predictions based on ski geometry. Two interesting application examples of integrative measurement setups that use combined interdisciplinary research approaches (such as biomechanics and physiology), are the study on the influence of sitting posture on the sit-skiing economy by Lajunen et al. and the study on the determinants of biathlon competition performance by Luchsinger et al. Finally, Steidl-Müller et al. investigated whether the relative age effect in youth competitive alpine skiing changed over the last decade; a comprehensive analysis with the data of more than 1,400 athletes, which benefits from today's digitalization in competitive sports.

In the context of injury/illness surveillance and prevention, the study by Doyle-Baker and Emery presented some interesting new data on physical activity, injury, and illness among adolescent skiers. Despite all technological advances and booming mHealth applications, trustworthy information provided by athletes and self-reported data from various stakeholders of the sport remains an important pillar for load management and injury registration. This also applies to the study by Westin et al. who evaluated a sports-specific anterior cruciate ligament (ACL) injury prevention programme based on a systematic injury monitoring system, installed at Swedish ski high schools. In their study, they reported a promising 45% reduction in the incidence rate of ACL injuries among young competitive alpine skiers when a prevention program consisting of neuromuscular exercise was implemented. Additionally, the review by Hanstock et al. aimed to provide an overview of pathophysiological responses to exercise at sub-zero temperatures and identify the potential of heat-and-moisture-exchanging breathing devices to prevent airway pathophysiological responses to cold air exercise.

In terms of return-to-sport, this Research Topic is complemented by the case study of Jordan et al. which presented a detailed analytical framework for return-to-sport training and neuromuscular testing used in an elite female alpine ski racer following ACL reconstruction. Interestingly, the authors reported that functional and strength deficits persisted up to 18 months post-surgery; a fact that further supports the use of an athlete monitoring approach that tracks them throughout the return-to-sport/return-to-performance transitions, rather than a discrete timepoint clearance approach. In this context, technology has great potential for the development of standardized and reliable neuromuscular assessment protocols and objective criteria.

## Where To Go From Here?

Given these general trends, new research articles, and the current state of scientific knowledge on health and performance assessment in winter sports, there are challenges that need to be addressed in the future.

### New Technologies and Approaches in General

- To be attractive for sports practitioners, new technologies and engineering solutions should be easy to use/calibrate and not hinder athletes with cumbersome multiple sensors, fixations, and connections. Ideally, sensors and electronics should be integrated or simply fixed into the athletes' standard equipment.
- However, industry and end-users must care about the objectivity, validity, and reliability of the technology applied. For effective decision-making in coaching and clinical practice, imprecise or even incorrect information can be worse than no additional information.

### Health and Performance Assessment

- New technologies and approaches should be used for providing evidence, not to confirm single observations and personal beliefs.
- A systematic and longitudinal collection of data is crucial. Here proceeding digitalization and technological development will help.
- Drawing practically relevant conclusions from a wealth of data *(information)* through data analytics *(knowledge)* will be one of our greatest challenges in the near future. In this context, the ongoing digitalization and new technological trends may provide significant support.
- An integrative fusion of different technologies and disciplines (e.g., biomechanics, physiology, psychology) will most likely become the new state-of-the-art in terms of health and performance assessment in winter sports.

## Author Contributions

JS, TS, and KA designed and edited this Research Topic. JS wrote the first draft of the corresponding editorial. All authors contributed to the manuscript and approved the final version.

## Conflict of Interest

The authors declare that the research was conducted in the absence of any commercial or financial relationships that could be construed as a potential conflict of interest.
